# The molecular, temporal and region-specific requirements of the beta isoform of Calcium/Calmodulin-dependent protein kinase type 2 (CAMK2B) in mouse locomotion

**DOI:** 10.1038/srep26989

**Published:** 2016-05-31

**Authors:** Martijn J. Kool, Jolet E. van de Bree, Hanna E. Bodde, Ype Elgersma, Geeske M. van Woerden

**Affiliations:** 1 Department of Neuroscience, Erasmus University Medical Center, 3015 CN, Rotterdam, The Netherlands; 2 ENCORE Expertise Center for Neurodevelopmental Disorders, Erasmus University Medical Center, 3015 CN, Rotterdam, The Netherlands

## Abstract

Genetic approaches using temporal and brain region-specific restricted gene deletions have provided a wealth of insight in the brain regions and temporal aspects underlying spatial and associative learning. However, for locomotion such extensive studies are still scarce. Previous studies demonstrated that *Camk2b*^–/–^ mice, which lack the β isoform of Calcium/Calmodulin-dependent protein kinase 2 (CAMK2B), show very severe locomotion deficits. However, where these locomotion deficits originate is unknown. Here we made use of novel *Camk2b* mutants (*Camk2b*^*f/f*^ and *Camk2b*^*T287*A^), to explore the molecular, temporal and brain region-specific requirements of CAMK2B for locomotion. At the molecular level we found that normal locomotion requires Calcium/Calmodulin mediated activation of CAMK2B, but CAMK2B autonomous activity is largely dispensable. At a systems level, we found that global deletion of *Camk2b* in the adult mouse causes only mild locomotion deficits, suggesting that the severe locomotion deficits of *Camk2b*^–/–^ mice are largely of developmental origin. However, early onset deletion of *Camk2b* in cerebellum, striatum or forebrain did not recapitulate the locomotion deficits, suggesting that these deficits cannot be attributed to a single brain area. Taken together, these results provide the first insights into the molecular, temporal and region-specific role of CAMK2B in locomotion.

The Calcium/Calmodulin-dependent protein kinase II (CaMKII, from hereon called CAMK2) family members are pivotal to normal synaptic plasticity and learning in the hippocampus, cortex and cerebellum. The family consists of four different isoforms, (alpha, beta, gamma and delta) of which CAMK2A is the most studied (for review, see^1^,^2^,^3^). In the last decade, the role of CAMK2B in the brain has gained attention largely due to the generation of new mutant mice^4^,^5^,^6^,^7^.

In sharp contrast to CAMK2A, CAMK2B has been shown to play an important role in locomotion, since two independent CAMK2B mutants both show severe locomotion deficits^4^,^6^. Like CAMK2A, CAMK2B is highly expressed in the brain^8^ and it has been shown that CAMK2B plays an enzymatic as well as a structural role in both hippocampal and cerebellar plasticity^4^,^5^. This structural role of CAMK2B comes from an additional domain within CAMK2B, the F-actin binding domain, which enables CAMK2B to cluster CAMK2A to the actin cytoskeleton^5^,^9^,^10^. Indeed, most hippocampal phenotypes observed in *Camk2b*^–/–^ mutants require CAMK2B protein, but not its enzymatic function, since the *Camk2b*^*A303R*^mutants, in which CAMK2B cannot be activated, do not show an overt hippocampal phenotype^5^. At the cerebellar parallel fiber–Purkinje cell synapse, CAMK2B appears to have both a structural role as an enzymatic role^4^ but it is unknown to what extend the severe locomotion deficits seen in *Camk2b*^–/–^ mice, arise from loss of the enzymatic or the structural role of CAMK2B.

Expression of CAMK2B starts around E12.5, whereas expression of CAMK2A starts postnatal, around P1^11^. According to this difference in temporal expression, one might expect that CAMK2B is involved in embryonic development, whereas CAMK2A is not. Indeed, it has been shown that deletion of CAMK2A in an adult brain gives a similar phenotype as germ-line deletion of CAMK2A with respect to learning and synaptic plasticity^12^. Similar studies have not been performed for CAMK2B, but considering the early onset of expression, it is conceivable that germ-line deletion of CAMK2B results in a more severe phenotype compared to deletion in adult animals.

Since CAMK2B has been shown to play an important role in controlling the direction of synaptic plasticity at the parallel fiber–Purkinje cell synapse^4^, the Purkinje cells are a likely candidate for being responsible for the locomotion deficits observed in the *Camk2b*^–/–^ mice. However, there are several other CAMK2B-expressing brain areas involved in motor control, such as motor cortex, several nuclei in brainstem, spinal cord and basal ganglia (e.g. striatum and subthalamic nucleus). Besides the hippocampus^5^,^13^, cerebellum^4^,^7^,^14^ and lateral habenula^15^, CAMK2B function in different specific motor control areas has not been studied. For this study we generated an autophosphorylation-deficient CAMK2B mutant (*Camk2b*^*T287A*^) and made use of the previously described CAMK2B mutant which can no longer bind Calcium/Calmodulin (*Camk2b*^*A303R*^)^5^ to study the structural and enzymatic role of CAMK2B in locomotion using the accelerating rotarod. Additionally, we generated a floxed *Camk2b* mutant to study the temporal and brain region-specific role of CAMK2B in motor behaviour. We show that Calcium/Calmodulin-dependent activation of CAMK2B is essential for normal locomotion, but surprisingly, CAMK2B autonomous activity is largely dispensable. Additionally, we found that normal locomotion requires CAMK2B to be present during development, and that the locomotion deficits observed in the *Camk2b* mutant cannot be assigned to a single brain area.

## Results

### The role of Calcium/Calmodulin-dependent and autonomous activity of CAMK2B in locomotion

We have previously shown that *Camk2b*^–/–^ mice (in which exon 11 was deleted) show severe locomotion deficits on the rotarod and the balance beam^4^. Since CAMK2 can have both structural and enzymatic functions in the brain, we first set out to understand which molecular aspects of CAMK2B are important for normal locomotion.

**Figure 1:**
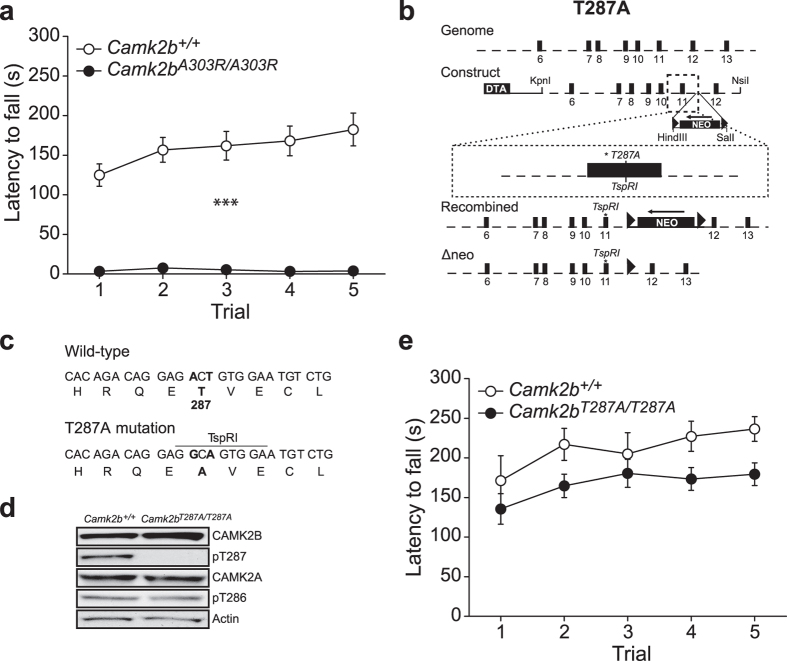
A crucial role for Calcium/Calmodulin-dependent activity, but not autonomous activity of CAMK2B in locomotion. Locomotion was tested using the accelerating rotarod. (**a**) *Camk2b*^*A303R/A303R*^mice (n = 6) show a severe impairment in locomotion compared to *Camk2b*^+/+^ control littermates (n = 14). (**b**) Schematic picture for the generation of the *Camk2b*^*T287A*^ mutants. Exons are depicted as black boxes. The asterisk in exon 11 indicates the mutation at Thr287, where a new TspRI restriction site was introduced. The *LoxP* sites flanking the neomycin gene are depicted as triangles. The Diphtheria Toxin cassette (DTA) was cloned in the construct for positive selection. Recombined depicts the mutant *Camk2b*^*T287A*^ locus after homologous recombination. ΔNEO depicts the mutant *Camk2b*^*T287A*^ locus after *Cre* recombination. (**c**) Sequence of Thr287 in exon 11 showing the specific mutation made to induce Thr287Ala and introducing the TspRI restriction site used for genotyping. (**d**) Western blots probed with a phospho-specific antibody against ph-T287 reveal no detectable Thr287 phosphorylation in the hippocampus of *Camk2b*^*T287A/T287A*^mice. Note that the Thr287Ala mutation has no effect on ph-Thr286, CAMK2A, and CAMK2B protein levels. Actin levels are shown as loading control. (**e**) *Camk2b*^*T287A/T287A*^mice (n = 14) show no impairment in locomotion compared to *Camk2b*^+/+^ control littermates (n = 9). Error bars indicate SEM.

Mutating Alanine 303 to Arginine (A303R) in CAMK2B, interferes with Calcium/Calmodulin binding and activation of CAMK2B, and renders CAMK2B into a persistently F-actin bound state^5^,^9^. To investigate whether this mutation has any effect on motor behaviour, we tested the *Camk2b*^*A303R/A303R*^ mice on the rotarod. Even though this mutant has normal hippocampal learning and plasticity^5^, *Camk2b*^*A303R/A303R*^ mice showed a severe locomotion deficit, not being able to stay on the rod for more than 5–10 seconds (effect of genotype: *F*_1,18_ = 39.05, p < 0.001; effect of time: *F*_1,18_ = 2.48, p = 0.05; interaction: *F*_1,18_ = 2.44, p = 0.05, repeated measures ANOVA; Fig. 1a).

The *Camk2b*^*A303R*^ mutation interferes with the Calcium/Calmodulin dependent activity, as well as the autonomous (Calcium/Calmodulin independent) activity of CAMK2B. To specifically investigate the role of autonomous activity in locomotion, we generated an autophosphorylation-deficient CAMK2B mouse mutant in which Threonine 287 is substituted by an Alanine, thus blocking autonomous CAMK2B activity (*Camk2b*^*T287A/T287A*^) (Fig. 1b,c). Protein quantification using western blot indeed revealed a near complete absence of T287-phosphorylated CAMK2B in *Camk2b*^*T287A/T287A*^mice without changes in T286-phosphorylated CAMK2A or total levels of both CAMK2A and CAMK2B (Fig. 1d; for quantification see Table 1).

*Camk2b*^*T287A/T287A*^ mice showed a trend towards reduced locomotion compared to their wildtype littermates, however this difference was not significant (effect of genotype: *F*_1,21_ = 3.72, p = 0.07; effect of time: *F*_1,21_ = 6.47, p < 0.001; interaction: *F*_1,21_ = 0.71, p = 0.59, repeated measures ANOVA; Fig. 1e). These results indicate that, in contrast to the Calcium/Calmodulin-dependent activation, the contribution of CAMK2B autonomous activity in normal locomotion is marginal.

### Temporal involvement of CAMK2B in locomotion

**Figure 2:**
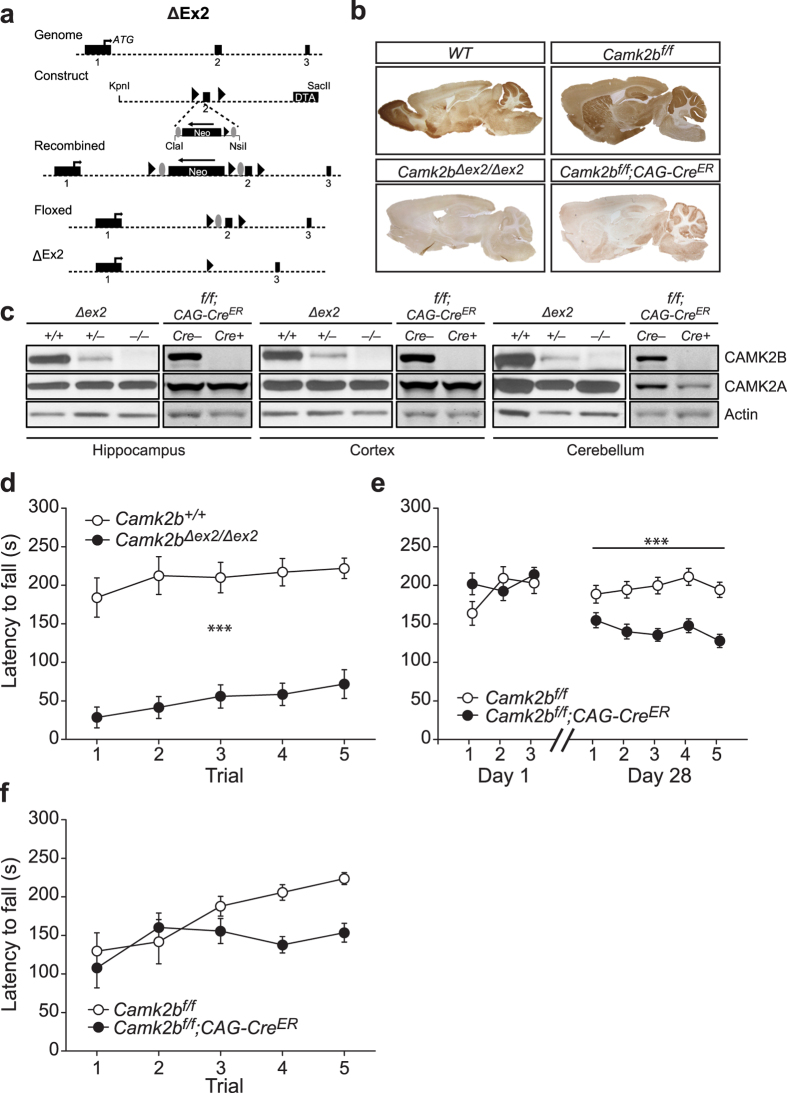
Temporal requirement of CAMK2B in locomotion. (**a**) Schematic overview of the generation of the floxed *Camk2b* and *Camk2b*^Δ*Ex2/*Δ*Ex2*^ mice. *Camk2b* locus and targeting construct with Exons 1, 2 and 3 depicted in black boxes. *LoxP* sites are indicated by the black triangles and the *Frt* sites are indicated by the grey ovals. The Diphtheria Toxin Cassette (DTA) was inserted for positive selection. Recombined depicts the mutant *Camk2b* locus after homologous recombination. Floxed depicts the *Camk2b*^*f/f*^ mutant locus after transient expression of the *Flp* recombinase, resulting in a floxed locus without the neomycin cassette. Δex2 depicts the *Camk2b*^Δ*Ex2*^ mutant locus after Cre-mediated deletion. (**b**) Immunohistochemistry stainings of CAMK2B, showing (Top to bottom, left to right): normal expression in *WT* mice and no expression in *Camk2b*^Δ*ex2/*Δ*ex2*^ mice; normal expression in *Camk2b*^*f/f*^mice and deletion throughout the brain in *Camk2b*^*f/f*^*;CAG-Cre*^*ER*^mice (**a**). (**c**) Western blots using an antibody specific for CAMK2A and CAMK2B in *Camk2b*^Δ*ex2/*Δ*ex2*^and *Camk2b*^*f/f*^*;CAG-Cre*^*ER*^mice and their control littermates. Actin levels are shown as loading control. Decreased levels of CAMK2B in *Camk2b*^*wt/*Δ*ex2*^ mice and no detection of CAMK2B in *Camk2b*^Δ*ex2/*Δ*ex2*^and *Camk2b*^*f/f*^*;CAG-Cre*^*ER*^ mice in hippocampus, cortex and cerebellum with no changes in levels of CAMK2A. (**d**) *Camk2b*^Δ*ex2/*Δ*ex2*^ mice (n = 8) show a significant impairment in locomotion compared to wildtype littermates (n = 8). (**e**) 8–10 week old *Camk2b*^*f/f*^*;CAG-Cre*^*ER*^mice (n = 13) and their *Camk2b*^*f/f*^ control littermates (n = 17) were trained on the rotarod before (Day 1) and 4 weeks after Tamoxifen injections (Day 28). Before deletion, both genotypes performed equally. After deletion, *Camk2b*^*f/f*^*;CAG-Cre*^*ER*^mice showed a significant impairment of locomotion compared to *Camk2b*^*f/f*^ control littermates as shown in the 5 trials given 28 days after the first injection. (**f**) 8–10 week old *Camk2b*^*f/f*^*;CAG-Cre*^*ER*^mice (n = 8) show no impairment in locomotion compared to *Camk2b*^*f/f*^ control littermates (n = 8) 4 weeks after Tamoxifen injections with no prior training. Error bars indicate SEM.

Expression of CAMK2B starts during early development (E12.5)^11^, hence the motor deficits seen in the *Camk2b*^–/–^ mice could very well be due to a crucial role for CAMK2B during development. To assess temporal contribution of CAMK2B in the severe locomotion deficits, we generated a novel floxed allele of *Camk2b*, with *LoxP* sites around exon 2 (containing the catalytic site) of the *Camk2b* gene (*Camk2b*^*f/f*^), which was crossed into the C57Bl/6 background (see Methods and Fig. 2a). To ensure that the locomotion deficits are also present in the C57Bl/6 background (the previous mutants were tested in an F2 129P2-C57Bl/6 hybrid background), we crossed the *Camk2b*^*f/f*^ mice with a *Cag-cre* transgene, deleting exon 2 from germline (*Camk2b*^Δ*ex2/*Δ*ex2*^). Using immunocytochemistry and western blots, we confirmed the global deletion of CAMK2B in this line. As expected, *Camk2b*^Δ*ex2/*Δ*ex2*^ showed loss of CAMK2B without changes in the expression of CAMK2A (Fig. 2b,c; Table 1). Furthermore, *Camk2b*^Δ*ex2/*Δ*ex2*^ mice showed a severe locomotion deficit compared to their wildtype littermates (effect of genotype: *F*_1,14_ = 48.01, p < 0.001, repeated measures ANOVA; Fig. 2d), which indicates that the motor deficits are present in both F2 129P2-C57Bl/6 hybrid mice^4^ as well as congenic (16 backcrosses) C57Bl/6 mice.

After having shown that our novel *Camk2b* mutant indeed recapitulates the severe locomotion deficit described before, we continued determining the developmental component of CAMK2B in the locomotion deficit. Therefore *Camk2b*^*f/f*^mice were crossed with *CAG-Cre*^*ER*^, giving us Tamoxifen-dependent temporal control over gene deletion. As expected, 12–14 week old *Camk2b*^*f/f*^*;CAG-Cre*^*ER*^ mice showed loss of CAMK2B 4 weeks after 4 daily consecutive injections with Tamoxifen and showed no changes in levels of CAMK2A (Fig. 2b,c; Table 1).

We tested 8–10 week old mice prior to Tamoxifen-induced deletion on the rotarod and found no difference between genotypes (effect of genotype: *F*_1,38_ = 0.53, p = 0.47, repeated measure ANOVA), indicating that before Tamoxifen mediated gene deletion, the *Camk2b*^*f/f*^*;CAG-Cre*^*ER*^ mice do not have a locomotion deficit (Fig. 2e*, left*). When tested 28 days after Tamoxifen-induced deletion, *Camk2b*^*f/f*^*;CAG-Cre*^*ER*^ mice showed a clear locomotion deficit on the accelerating rotarod (effect of genotype: *F*_1,38_ = 24.25, p < 0.001, repeated measures ANOVA; Fig. 2e, *right*), however, this phenotype was not as severe as upon germ-line deletion. To make sure that the milder phenotype was not caused by the initial rotarod testing before gene deletion, we induced *Camk2b* gene deletion in a naïve cohort of 8–10 week old mice, and tested the mice 4 weeks after gene deletion. Although these *Camk2b*^*f/f*^*;CAG-Cre*^*ER*^ mice showed no improvement of locomotion over time compared to their *Camk2b*^*f/f*^ littermates without expression of CRE (overall effect of genotype: *F*_1,14_ = 4.01, p = 0.06; effect of time: *F*_1,14_ = 6.48, p < 0.001; interaction: *F*_1,14_ = 3.02, p < 0.05 Fig. 2f), performance at the first two trials was indistinguishable from control mice, which is markedly different from *Camk2b*^Δ*ex2/*Δ*ex2*^ mice (Fig. 2d)

Taken together, deletion of CAMK2B in adulthood resulted in a much milder locomotion deficit compared to germline deletion of CAMK2B, indicating a significant developmental origin for the locomotion deficits seen in *Camk2b*^Δ*ex2/*Δ*ex2*^ mice.

### Brain areas contributing to the CAMK2B dependent rotarod deficits

Lesion studies in rodents have indicated that several brain areas are involved in locomotion, the most important being the cerebellum, striatum and motor cortex (for review see^16^). To our knowledge, the specific contribution of each of these brain areas to rotarod motor behaviour has not been systematically assessed through genetic lesions. CAMK2B is expressed in all these brain regions, therefore we used our conditional mutant to assess which brain area, if not all, is responsible for the severe rotarod phenotype seen in *Camk2b*^Δ*ex2/*Δ*ex2*^ mice. Knowing that loss of CAMK2B in the cerebellum reverses plasticity at the parallel fiber–Purkinje cell synapse, we started with assessing the importance of the cerebellum in the rotarod phenotype. *Camk2b*^*f/f*^mice were crossed with two different cerebellum-specific *Cre*-lines: *L7-cre* (specific for cerebellar Purkinje cells) and *Gabaa6-cre* (specific for cerebellar granule cells). Both *Cre*-lines express the CRE protein within the first postnatal week^17^,^18^.

**Figure 3:**
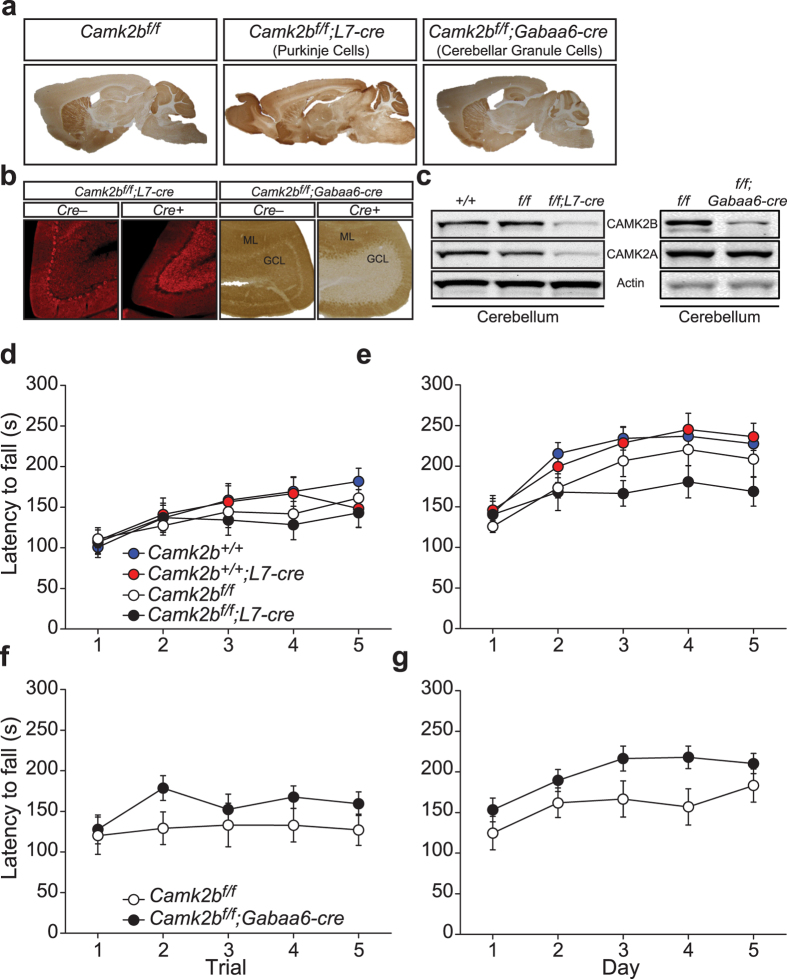
Requirement of cerebellar CAMK2B in locomotion. Motor performance (1-day paradigm; (**d**,**f**)) and learning (5-day paradigm; (**e**,**g**)) was tested using the accelerating rotarod. (**a**) Immunohistochemistry stainings of CAMK2B, showing (left to right): normal expression in *Camk2b*^*f/f*^mice; specific deletion in cerebellar Purkinje cells in *Camk2b*^*f/f*^*;L7-cre* mice and specific deletion in the cerebellar granule cells of *Camk2b*^*f/f*^*;Gabaa6-cre* mice. (**b**) Immunofluorescent and immunohistochemical zoomed-in picture of the cerebellum of *Camk2b*^*f/f*^mice, *Camk2b*^*f/f*^*;L7-cre* and *Camk2b*^*f/f*^*;Gabaa6-cre* mice showing specific deletion in cerebellar Purkinje Cells in the *Camk2b*^*f/f*^*;L7-cre* mice and deletion in the granule cell layer (GCL) of *Camk2b*^*f/f*^*;Gabaa6-cre* mice, but not in Purkinje cells of the molecular layer (ML). (**c**) Western blots using an antibody specific for CAMK2A and CAMK2B in the different *Camk2b* mutants and their control littermates. (Left) Decreased levels of CAMK2A and CAMK2B in the cerebellum of *Camk2b*^*f/f*^*;L7-cre* mice. *LoxP* sites do not affect CAMK2A and CAMK2B levels. (Right) Decreased levels of CAMK2B in the cerebellum of *Camk2b*^*f/f*^*;Gabaa6-cre* mice with no changes in CAMK2A levels. Actin levels are shown as loading control. (**d**) *Camk2b*^*f/f*^*;L7-cre* mice (n = 10) show no difference in performance compared to *Camk2b*^*f/f*^ control littermates (n = 12). *Camk2b*^+/+^*;L7-cre* (n = 12) and *Camk2b*^+/+^ mice (n = 15) do not differ in performance compared to *Camk2b*^*f/f*^*;L7-cre* and *Camk2b*^*f/f*^mice implying no effect of CRE or the flanked *LoxP* sites on rotarod performance. (**e**) *Camk2b*^*f/f*^*;L7-cre* mice (n = 10) show an impairment in learning compared to *Camk2b*^*f/f*^ control littermates (n = 12). *Camk2b*^+/+^*;L7-cre* (n = 12) and *Camk2b*^+/+^ mice (n = 15) do not differ in learning compared to *Camk2b*^*f/f*^*;L7-cre* and *Camk2b*^*f/f*^mice implying no effect of CRE or the flanked *LoxP* sites on rotarod learning. (**f**) *Camk2b*^*f/f*^*;Gabaa6-cre* mice (n = 8) show no impairment in performance compared to *Camk2b*^*f/f*^ control littermates (n = 8). (**g**) *Camk2b*^*f/f*^*;Gabaa6-cre* mice (n = 8) and *Camk2b*^*f/f*^ control littermates (n = 8) show normal learning. Error bars indicate SEM.

*Camk2b*^*f/f*^*;L7-cre* mice showed selective loss of CAMK2B in cerebellar Purkinje cells (Fig. 3a–c; Table 1) and *Camk2b*^*f/f*^*;Gabaa6-cre* mice showed selective loss of CAMK2B in cerebellar granule cells. Surprisingly, *Camk2b*^*f/f*^*;L7-cre* mice also showed a decrease in levels of CAMK2A in the cerebellum, whereas in *Camk2b*^*f/f*^*;Gabaa6-cre* mice CAMK2A levels were unaffected (Fig. 3a,c; Table 1). Importantly, *Camk2b*^*f/f*^ mice lacking CRE recombinase did not show any detectible reduction of CAMK2B protein compared to *Camk2b*^+/+^ mice, indicating that the insertion of the *LoxP* sites did not interfere with *Camk2b* gene expression (Table 1).

For the rotarod experiments with the *L7-cre* mice, we used all four genotypes obtained from crossings of heterozygous *Camk2b*^*f/*+^ (with and without cre) mice: *Camk2b*^*f/f*^*;L7-cre, Camk2b*^*f/f*^*, Camk2b*^+/+^ and *Camk2b*^+/+^*;L7-cre*. Finding no difference in locomotion between the *Camk2b*^+/+^ and *Camk2b*^*f/f*^ mice, we decided for subsequent experiments to cross homozygous *Camk2b*^*f/f*^ mice (with and without cre), resulting in only *Camk2b*^*f/f*^ and *Camk2b*^*f/f*^*;cre* offspring. Surprisingly, neither the cerebellar Purkinje cell nor granule cell specific *Camk2b* mutants showed a rotarod deficit on the one-day training paradigm (Fig. 3d,f; for statistics see Table 2). When tested for 5 consecutive days however (to assess motor learning), *Camk2b*^*f/f*^*;L7-cre* mice showed impaired motor learning (Fig. 3e; Table 2), whereas the granule cell specific mutants still showed no hint of any deficit compared to their littermate control mice (Fig. 3g; Table 2).

**Figure 4:**
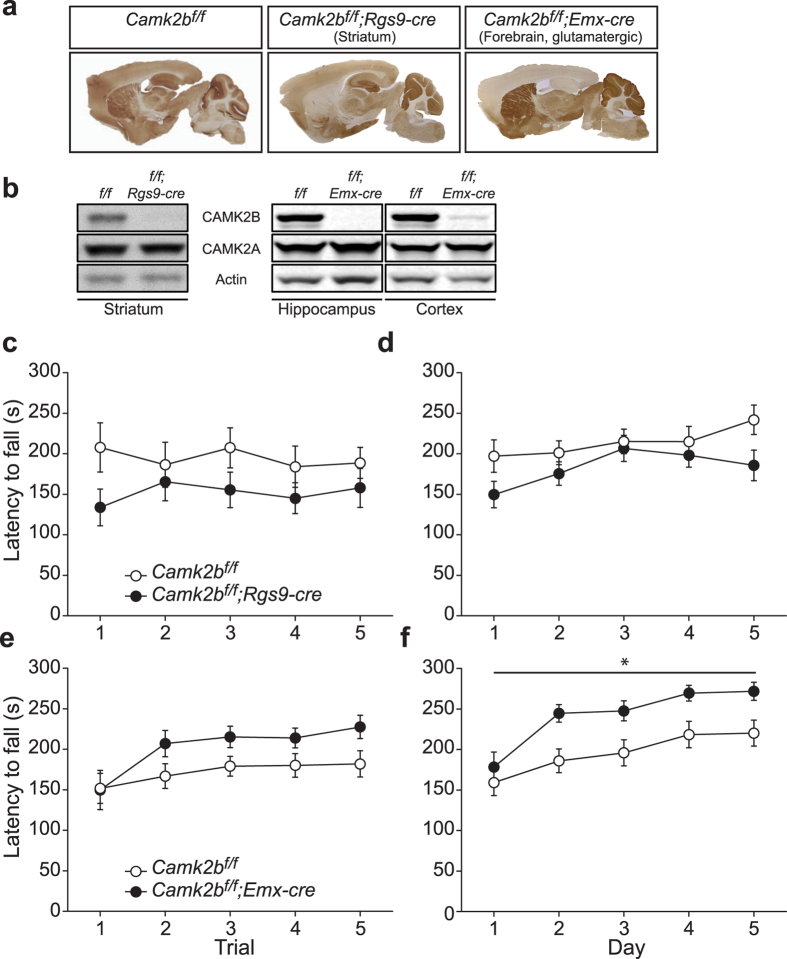
Requirement of striatal, hippocampal and cortical CAMK2B in locomotion. Motor performance (1-day paradigm; (**c**,**e**)) and learning (5-day paradigm; (**d**,**f**)) was tested using the accelerating rotarod. (**a**) Immunohistochemistry stainings of CAMK2B, showing (left to right): normal expression in *Camk2b*^*f/f*^mice; specific deletion in the striatum of *Camk2b*^*f/f*^*;Rgs9-cre* and specific hippocampal and cortical deletion in *Camk2b*^*f/f*^*;Emx-cre* mice. (**b**) Western blots using an antibody specific for CAMK2A and CAMK2B in the different *Camk2b* mutants and their control littermates. Actin levels are shown as loading control. Decreased levels of CAMK2B in striatal tissue of *Camk2b*^*f/f*^*;Rgs9-cre* mice (left). Decreased hippocampal and cortical levels of CAMK2B in *Camk2b*^*f/f*^*;Emx-cre* mice (right). No changes in levels of CAMK2A were observed in both mutants. (**c**) *Camk2b*^*f/f*^*;Rgs9-cre* mice (n = 7) show no impairment in performance compared to *Camk2b*^*f/f*^ control littermates (n = 7). (**d**) *Camk2b*^*f/f*^*;Rgs9-cre* mice (n = 7) and *Camk2b*^*f/f*^ control littermates (n = 7) show normal learning. (**E**) *Camk2b*^*f/f*^*;Emx-cre* mice (n = 13) show no impairment in performance compared to *Camk2b*^*f/f*^ control littermates (n = 17). (**f**) *Camk2b*^*f/f*^*;Emx-cre* mice (n = 13) and *Camk2b*^*f/f*^ control littermates (n = 17) show enhanced performance but no impairment in learning. Error bars indicate SEM.

Since the loss of CAMK2B in cerebellar granule cells or Purkinje cells does not appear to contribute to the locomotion deficit as seen in *Camk2b*^Δ*ex2/*Δ*ex2*^ mice, we then focused on striatum and motor cortex. *Camk2b*^*f/f*^ mice were crossed with *Rgs9-cre* (deletion specifically in most medium spiny projection neurons of the striatum^19^) and *Emx-cre* (deletion in glutamatergic pyramidal neurons in cortex and hippocampus) transgenic mice to test respectively striatal and cortical involvement in the rotarod phenotype. *Rgs9-cre* is expressed from P8 onwards^20^ and *Emx-cre* is expressed from E10.5^21^. *Camk2b*^*f/f*^*;Rgs9-cre* mice showed selective loss of CAMK2B in striatum (Fig. 4a,b; Table 1) and *Camk2b*^*f/f*^*;Emx-cre* mice showed selective loss of CAMK2B in glutamatergic pyramidal neurons in hippocampus and cortex with no changes in levels of CAMK2A in both mutants (Fig. 4a,b; Table 1). Importantly, none of the specific lines showed notable off-target deletion in other brain areas (data not shown).

Notably, neither the striatal nor forebrain-specific *Camk2b* mutants showed a rotarod deficit on the one-day training paradigm (Fig. 4c,e; for statistics see Table 2). When tested for 5 consecutive days these region-specific mutants still showed no deficits compared to their controls (Fig. 4d,f; Table 2). Interestingly, the *Camk2b*^*f/f*^*;Emx-cre* mice even showed significantly enhanced locomotion (Fig. 4f; Table 2).

Taken together these results show that selective deletion of CAMK2B in brain areas supporting locomotion, is not sufficient to recapitulate the locomotion deficits observed in either the *Camk2b*^Δ*ex2/*Δ*ex2*^ or the *Camk2b*^*f/f*^*;CAG-Cre*^*ER*^ mice. This suggests that multiple brain areas are responsible for locomotion deficits observed in *Camk2b*^Δ*ex2/*Δ*ex2*^ mice. The only mouse line showing a small but significant effect on motor learning (5 day paradigm), but not motor performance (1 day paradigm), was the Purkinje cell specific knockout (*Camk2b*^*f/f*^*;L7-cre*). However, this effect is still marginal compared to *Camk2b*^Δ*ex2/*Δ*ex2*^ mice.

## Discussion

Using several novel as well as previously described *Camk2b* mouse mutants, we dissected the molecular, temporal and systems requirements for CAMK2B in locomotion. At the molecular level we showed that even though the autonomous activity of CAMK2B in locomotion is dispensable, the ability of CAMK2B to bind Calcium/Calmodulin is crucial for normal locomotion. At the developmental level we found that deletion of *Camk2b* in the adult mouse causes a much milder locomotion deficit compared to germ line *Camk2b* deletion. Finally, at a systems level we showed that early onset deletion of *Camk2b* in any of the major motor areas of the brain (cerebellum, striatum or forebrain) did not recapitulate the locomotion deficit of global *Camk2b* deletion. Taken together these results suggest that the locomotion deficits are mainly of a developmental origin, as well as a result of interplay between multiple regions (systems level).

We showed here that loss of autophosphorylation of CAMK2B at the T287 site does not significantly affect rotarod behaviour, although a small trend could be seen. In contrast, mutating the Calcium/Calmodulin binding site of CAMK2B, such that it cannot bind Calcium/Calmodulin and rendering CAMK2B bound to actin (*Camk2b*^*A303R/A303R*^), led to a severe locomotion deficit. Whether the inability to bind Calcium/Calmodulin and becoming active, or the inability to be released from actin causes the deficits observed, remains to be investigated. CAMK2B forms heteromers with CAMK2A^22^ and ratios of CAMK2A/CAMK2B can differ between cell types^22^,^23^. It has been shown for the hippocampus (in dissociated neuronal cultures and using *Camk2b*^*A303R/A303R*^ mice) that CAMK2B kinase activity is not important for synaptic plasticity or hippocampal learning, but that CAMK2B is required for regulating the subcellular location of CAMK2A^5^,^9^. However, in Purkinje cells, CAMK2B not only regulates the subcellular localization of CAMK2A, but also plays an important enzymatic role in regulating the direction of plasticity at the parallel fiber–Purkinje cell synapse^4^. These different requirements likely reflect the differences in the CAMK2A/CAMK2B ratio in the different brain areas. Thus, when the relative expression level of CAMK2A is low, both the enzymatic and the non-enzymatic function of CAMK2B may be important, whereas in areas where CAMK2A is abundant, CAMK2B serves mainly to regulate the subcellular localization of CAMK2A. It would be interesting to investigate the CAMK2A/CAMK2B ratio in the specific motor areas of the brain to get more insight in which brain areas are involved in the severe locomotion deficits seen in the *Camk2b*^–/–^ and *Camk2b*^*A303R/A303R*^ mice. Notably, *Camk2b*^*f/f*^*;L7-cre* mice showed a significant decrease in CAMK2A in western blot when only CAMK2B was deleted. A similar trend could be found in the cerebellum of *Camk2b*^*f/f*^*;CAG-Cre*^*ER*^ and in Camk2b^–/–^ mice^4^. These results imply that specifically in Purkinje cells, which are the only cells in the cerebellum expressing CAMK2A, CAMK2B stabilizes CAMK2A, and that absence of CAMK2B therefore results in reduced levels of CAMK2A. However, the exact mechanism behind this regulation requires further research.

From a developmental point of view, we found that deleting most of *Camk2b* in the adult mice resulted in a significant locomotion impairment showing its necessity for normal motor behaviour. However, the phenotype was rather mild compared to the germ-line knock out mouse, indicating that acute deletion of *Camk2b* does not dramatically affect neuronal function. These results indicate that the severe locomotion deficits of *Camk2b*^–/–^ mice largely arise during development of the nervous system. Unfortunately, little is known about the role of both the CAMK2 isoforms in brain development. For CAMK2A, which starts to be expressed around P1, we recently showed that deletion of *Camk2a* in adulthood is as detrimental as a germline deletion of *Camk2a* for hippocampal learning and plasticity^12^, indicating an important post-developmental role. CAMK2B however, starts to be expressed already during early embryonic development (around E12.5)^11^,^24^. Therefore it is likely that CAMK2B plays a significant role during development. Previous studies looking at the developmental role of CAMK2B have shown an important role for CAMK2B in neurite extension and spine formation in acute knock-down experiments in neuronal cultures^25^. However, no morphological changes were found in the *Camk2b*^–/–^ mice^4^,^6^, nor in neuronal cultures obtained from *Camk2b*^–/–^ mice^5^, thus it is unclear how a germ-line deletion of *Camk2b* can be so profoundly affected.

Locomotion deficits can be caused by dysfunction of several different brain areas (as reviewed by^16^). Using lesion studies it was shown that the cerebellum and thalamus are important in rotarod behaviour^26^,^27^,^28^, but lesioning the dorsal striatum did not cause motor impairments on the rotarod^29^. As for the motor cortex, some lesion studies have been performed, but the effect of the lesions was not assessed by the rotarod task. However, a recent pharmacological study showed that mGluR5 inhibition in primary motor cortex affected rotarod performance, which was not observed upon mGluR5 inhibition in dorsolateral striatum^30^. In contrast, deletion of the *Nmdar1* gene from P8 onwards using *Rgs9-cre* mice in the striatum resulted in performance and learning deficits on the rotarod^20^. Taken together, these studies all indicate that, depending on the lesion or genetic change, several specific brain areas could potentially be responsible for deficits in motor performance as measured with the rotarod.

Surprisingly, restricted deletion of CAMK2B in the major motor areas of the brain did not reveal a specific brain area or cell type to be responsible for the severe rotarod phenotype observed in the *Camk2b*^–/–^ mice. In these groups we not only assessed motor performance, but also motor learning by testing the mice over 5 consecutive days. Even though there are small variations in performance and learning in the control groups between the different experiments, the effects of *Camk2b* deletion on locomotion was only marginal compared to the profound phenotypes seen in *Camk2b*^–/–^, *Camk2b*^Δ*ex2/*Δ*ex2*^and *Camk2b*^*A303R/A303R*^ mice. There are three possible explanations for our findings. First, onset of *Camk2b* gene deletion in our mutants is likely to be too late. Three of the *cre*-lines used in this study start *cre* expression after onset of CAMK2B expression (*L7-cre* starts expression at P1^18^, *Gabaa6-cre* starts expression at P5-P7^17^, and *Rgs9-cre* expression is seen as early as P8^20^). Thus, these lines start off with normal embryonic CAMK2B expression. Since we do not know the critical time window for CAMK2B in rotarod behaviour, it could be that in these mice CAMK2B is deleted too late in these separate brain regions or cell types to cause a significant effect on rotarod behaviour, comparable to *Camk2b*^*f/f*^*;CAG-Cre*^*ER*^mice, where adult deletion resulted only in a mild phenotype when CAMK2B was deleted throughout the brain. Only the *Emx-cre* line, which expresses *cre* from E10.5^21^, induces deletion of *Camk2b* before onset of CAMK2B expression (E12.5). Interestingly, deletion in this *cre*-line resulted in enhanced, instead of impaired performance. Second, *Camk2b* deletion in thalamus, brainstem and the deep cerebellar nuclei is only obtained in the *Camk2b*^Δ*ex2/*Δ*ex2*^ mice. Since it has been shown that these areas are involved in locomotion^16^, there is a possibility that the motor deficits arise from those areas. Third, the severity of the locomotion deficits observed in the *Camk2b*^–/–^ mice^4^ could indicate a distributed role for CAMK2B in several brain areas and cell types, each one of them contributing a little to the deficit. For example, we cannot exclude that deleting *Camk2b* in Purkinje-cells and cerebellar granule cells simultaneously would have an effect on locomotion. Additionally, even though the different *cre*-lines used in this study were very effective at deleting *Camk2b* throughout multiple brain areas and cell types (as judged by immunuhistochemstry and Western blot), we cannot exclude the possibility that a small number of cells did not undergo deletion, and prevented the lack of a severe phenotype.

Taken together we conclude that the nature of severe the locomotion deficits observed in the *Camk2b*^–/–^ is predominantly of developmental origin and cannot be attributed to one specific motor area or cell type in the brain. Moreover we conclude that the Calcium/Calmodulin-dependent activation of CAMK2B is essential for normal locomotion, but that the autonomous activity of CAMK2B is largely dispensable for normal locomotion. These findings are in sharp contrast to the role of CAMK2A in spatial learning, where loss of CAMK2A in adult mice recapitulates the phenotype of *Camk2a*^–/–^ mice, where selective loss of CAMK2A in the hippocampus causes spatial learning deficits, and where loss of autonomous CAMK2A activity causes severe spatial learning deficits^12^,^31^.

## Materials and Methods

### Animals

*Camk2b*^*A303R/A303R*^ *and Camk2b*^*T287A/T287A*^ mice were tested in a 129P2-C57Bl/6OlaHsd F2 hybrid background. *Camk2b*^Δ*ex2/*Δ*ex2*^mice were backcrossed >16 times into C57Bl/6JOlaHsd background. All conditional mice used in this study were backcrossed 10–12 times into the C57BL/6JOlaHsd background and crossed with cre lines maintained in the C57BL/6JOlaHsd background. Mice were genotyped when they were 7–10 days old, and re-genotyped after the mice were sacrificed. Genotyping records were obtained and kept by a technician not involved in the experimental design, performance and analysis. All mice were tested between 2–3 months of age, except the *Camk2b*^*f/f*^*;Rgs9-cre* group, which was tested between 3–5 months of age. All mice were kept group-housed in IVC cages (Sealsafe 1145 T, Tecniplast) with bedding material (Lignocel BK 8/15 from Rettenmayer) on a 12/12 h light/dark cycle in 21 °C (±1 °C), humidity at 40–70% and with food pellets (801727CRM(P) from Special Dietary Service) and water available *ad libitum*. For all experiments mutants were compared to WT or *cre*-negative homozygous floxed littermates. All groups were matched for age and sex and all experiments were done during daytime. Experimenters were blind for genotype throughout experiments and data analysis. All research was performed in accordance with and approved by a Dutch Animal Ethical Committee (DEC) for animal research.

### Generation of Camk2b^A303R/A303R^ and Camk2b^T287A/T287A^ mice

Generation of the *Camk2b*^*A303R/A303R*^ mice has been described previously^5^. The *Camk2b*^*T287A*^ targeting construct to generate *Camk2b*^*T287A/T287A*^ *mice* was generated as follows. The *Camk2b* genomic sequence (ENSMUSG00000057897) was obtained from a public database (Ensembl) and used to design the primers for the targeting constructs. PCR fragments encompassing exon 6–11 using 5′ primer: 5′-GGTACCTGAGGAAGGTGCCAGCTCTGTCCC-3′ and 3′ primer: 5′–GTCGACCAGGGTAGTCACGGTTGTCC-3′ (5.3 Kb; exon denotation according to ENSMUST00000019133) and exon 11–12 using 5′ primer: 5′-GCGGCCGCCTGTTAAAGGAATGGTTCTC-3′ and 3′ primer: 5′-ATGCATCTAAAAGGCAGGCAGGATGATCTGC-3′ (6 Kb) were amplified using High Fidelity Taq Polymerase (Roche) on ES cell genomic DNA and cloned on either site of a PGK-Neomycin selection cassette. All exons were verified by sequencing. Site directed mutagenesis was used to introduce the Thr287Ala point mutation, which induced a TspRI restriction site (Fig. 1). For counter selection, a gene encoding Diphtheria toxin chain A (DTA) was inserted at the 5′ end of the targeting construct. The targeting construct was linearized and electroporated into E14 ES cells (derived from 129P2 mice). Cells were cultured in BRL cell conditioned medium in the presence of Leukaemia inhibitory factor (LIF). After selection with G418 (200 μg/ml), targeted clones were identified by PCR (long-range PCR from Neomycin resistance gene to the region flanking the targeted sequence). A clone with normal karyotype was injected into blastocysts of C57Bl/6 mice. Male chimeras were crossed with female C57Bl/6 mice (Harlan). The resulting F1 heterozygous mice (in the 129P2-C57Bl/6 background) were subsequently inter-crossed to obtain F2 129P2-C57Bl/6 hybrid mice to generate homozygous mutants and wild-type littermate controls.

### Generation of floxed Camk2b and Camk2b^Δex2/Δex2^ mice

The floxed *Camk2b* targeting construct was generated as follows. The *Camk2b* genomic sequence (ENSMUSG00000057897) was obtained from a public database (Ensembl) and used to design the primers for the targeting constructs. PCR fragments encompassing intron 1 using 5′ primer: 5′-TTTGGTACCGCATTGTGGGCATCTATGAAG-3′ and 3′ primer: 5′–AAAGGATCCAGTCAGCTGGAATGAGACGTG-3′ (2.6 Kb; intron and exon denotation according to ENSMUST00000019133), exon 2 using 5′ primer: 5′-TTTATGCATGATAGACCTGGGTGTTACACAG-3′ and 3′ primer: 5′-AAAGTCGACCTTCAGGTCTGGGACAGAG-3′ (583 bp) and intron 2 using 5′ primer: 5′-TTTGCGGCCGCAGAAGTCCTCATATTGGGGAGG-3′ and 3′ primer: 5′-AAACCGCGGTGACTCCTAATGCAGAAGACACC-3′ (4 kb) were amplified using High Fidelity Taq Polymerase (Roche) on ES cell genomic DNA. The 5′ fragment containing part of intron 1 was inserted before the first *LoxP* site whereas exon 2 and part of intron 2 were cloned on either site of a PGK-Neomycin selection cassette, which is flanked by *frt* and *LoxP* sites (for the schematics see Fig. 2). Exon 2 and flanking intronic sequences were sequenced to verify that the absence of secondary mutations. For counter selection, a gene encoding Diphtheria toxin chain A (DTA) was inserted at the 5′ of the targeting construct. The targeting construct was linearized and electroporated into E14 ES cells (derived from 129P2 mice). Cells were cultured in BRL cell conditioned medium in the presence of Leukaemia inhibitory factor (LIF). After selection with G418 (200 μg/ml), targeted clones were identified by PCR (long-range PCR from Neomycin resistance gene to the region flanking the targeted sequence). To delete the Neomycin resistance cassette and obtain the conditional construct, the correctly targeted clones were transiently transfected with an *flp* recombinase (Fig. 2). Finally, a clone with correct karyotype was injected into blastocysts of C57Bl/6 mice. Male chimeras were crossed with female C57BL/6JOlaHsd mice (Harlan). The resulting F1 heterozygous *Camk2b*^*f/*+^ mice, which were backcrossed 10–12 times with C57BL/6JOlaHsd mice before generating the brain region and temporally restricted mutants as described below. To obtain the *Camk2b*^Δ*ex2/*Δ*ex2*^ mice, the *Camk2b*^*f/*+^ mice were crossed with transgenic *Cag-cre* mice^32^. The *Camk2b*^Δ*ex2/*+^ heterozygous offspring were backcrossed >16 times in C57BL/6JOlaHsd to obtain a congenic line.

### Generation of Camk2b^*f/f*^ conditional mutants

For the generation of conditional mutants, female *Camk2b*^*f/f*^ mice (backcrossed 10–12 times with C57BL/6JOlaHsd) were crossed with male transgenic Cre lines maintained in C57BL/6JOlaHsd. To obtain temporal control, *Camk2b*^*f/f*^ mice were crossed with *CAG-Cre*^*ER*^ mice (RRID:IMSR_JAX:004682^33^). F1 heterozygous floxed female *Camk2b*^*f/*+^ mice were then crossed with male heterozygous floxed mice expressing *CAG-Cre*^*ER*^ to obtain the desired F2 genotypes: homozygous floxed *Camk2b*^*f/f*^*;CAG-Cre*^*ER*^ with transgenic expression of *CAG-Cre*^*ER*^ (mutants) and *Camk2b*^*f/f*^ without *CAG-Cre*^*ER*^ expression (controls). A similar crossing was performed to obtain Purkinje cell specific deletion of CAMK2B, using *L7/pcp-2 cre* transgenic mice (RRID:IMSR_JAX:004146^18^,^34^, and to obtain glutamatergic and telencephalic-restricted deletion of CAMK2B, using *Emx1-cre* transgenic mice (RRID:IMSR_RBRC01345^21^). Deletion of *Camk2b* in cerebellar granule cells was obtained by crossing *Camk2b*^*f/f*^ mice with *Gabaa6-cre* transgenic mice^17^. To generate striatal specific deletion of CAMK2B we crossed *Camk2b*^*f/f*^ mice with *Rgs9-cre* mice^20^.

### Tamoxifen injections

Adult *Camk2b*^*f/f*^ and *Camk2b*^*f/f*^*;CAG-Cre*^*ER*^mice (8–10 weeks of age) were injected with Tamoxifen intraperitoneally (Sigma-Aldrich) (0.1mg/gr of bodyweight) for 4 consecutive days. To keep the dose of Tamoxifen constant throughout injection days we kept a tight injection scheme, injecting mice 24+/−1 hour after the previous injection. Tamoxifen was dissolved in sunflower oil (20 mg/ml). Behavioural testing was assessed 4 weeks after the first injection. Even though Tamoxifen does not have an effect on emotional reactivity, neurological functioning or learning^35^ we injected both *Camk2b*^*f/f*^ and *Camk2b*^*f/f*^*;CAG-Cre*^*ER*^ mice to control for any possible effects of Tamoxifen.

### Rotarod

The accelerating rotarod (Ugo Basile, Comerio Varese, Italy, 7650) contains a cylinder 3 cm in diameter and can train 5 mice at the same time. Rotarod speed starts at 4 r.p.m., which increases to 40 r.p.m. at 270 seconds. The experiment stopped at 300 seconds. Latency to fall was measured in seconds after a mouse (i) fell of, (ii) clung to the rod for 3 consecutive rotations or (iii) clung to the rod for 2 rotations twice within 10 seconds. Mice were trained with an inter-trial interval of 45 minutes. We used two different paradigms on the accelerating rotarod. For motor performance (which we define as locomotion throughout the text) we used 5 consecutive trials with naïve mice (1 day paradigm). To assess motor learning we took the average of the first two trials of the first day and continued training the mice for another 4 consecutive days with 2 trials per day (5 day paradigm).

### Immunohistochemistry and immunofluorescence

Mice were anaesthetized with pentobarbital and perfused transcardially with PBS followed by freshly prepared 4% paraformaldehyde solution (PFA, Sigma). Brains were taken out after perfusion and post-fixed for 1.5 hours in PFA and afterwards kept in 30% sucrose solution overnight. Immunohistochemistry was performed on free-floating 40μm thick sagittal cryostat sections. Sections were washed in PBS once and afterwards primary antibodies were added (anti-CAMK2B, 1:2000, #ab34703, Abcam) diluted in PBS containing 2% NHS, 0.5% Triton-X 100 and 150 mM bovine serum albumin (BSA) and kept at 4 °C overnight for 48 hours. Two days later sections were washed 3 times with PBS and then secondary antibodies were added (biotinylated goat anti-rabbit IgG antibody, Vector Laboratories, Burlinghame, CA; 1:200) diluted in PBS containing 2% NHS, 0.5% Triton-X 100 and 150 mM bovine serum albumin (BSA) for 1 to 2 hours on room temperature. For diaminobenzidine (DAB) staining, sections were processed using a standard avidin-biotin-immunoperoxidase complex method (ABC, Vector Laboratories, USA) and 0.05% DAB as the chromogen. Sections were mounted on slices using chrome(3)potassiumsulfatedodecahydrate and left to dry. The next day, slides were dehydrated in alcohol, cleared with xylene and covered using Permount (Fisher Scientific, USA). For immunofluorescence, the same CAMK2B was used as in immunohistochemistry (1:1000) and we used Cy3 rabbit (1:200) as a secondary antibody. After 1–2 hours incubation of the secondary antibody at room temperature sections were washed four times in PB (0.05 M) and mounted on slices using chrome(3)potassiumsulfatedodecahydrate and left to dry. Finally, sections were covered using Mowiol (Sigma-Aldrich).

### Western blot

Mice were anaesthetized using isoflurane and sacrificed by decapitation. Brain samples were taken out quickly and stored in liquid nitrogen. Upon protein determination lysates were first prepared and brain samples were homogenized in lysis buffer (10 mM Tris-HCl 6.8, 2.5% SDS, 2 mM EDTA). Protein concentration in the samples was determined and then lysate concentrations were adjusted to 1 mg/ml. Western blots were probed with primary antibodies against either CAMK2A (6G9, 1:40.000, Abcam), CAMK2B (CB-β1, 1:10.000, Invitrogen), Actin (MAB1501R, 1:20.000, Chemicon) or Ph-T286/T287 (autophosphorylated *α* CAMK2A and CAMK2B antibody; #06–881; 1:5000; Upstate Cell Signaling Solutions) and secondary antibodies (goat anti-mouse and/or goat anti-rabbit, both 1:3000, AffiniPure #115-007-003 and #111-007-003). Blots were stained with Enhanced ChemoLuminescence (ECL) (#32106, Pierce) or stained and quantified using LI-COR Odyssey Scanner and Odyssey 3.0 software. Quantification of western blot in ECL was done using ImageJ.

### Data analysis and statistics

All experiments and analyses were performed blind to genotype. All behavioural tests were analysed using a 2-WAY repeated measures ANOVA to determine the performance and learning of the genotypes, with genotype as the between subjects factor and the repeated measures as within subject factor. α was set at 0.05. All values represent average+/− SEM. Group sizes for each genotype are depicted in the figure legends. All statistics were performed in Graphpad Prism. *p < 0.05, ***p < 0.001.

## Additional Information

**How to cite this article**: Kool, M. J. *et al.* The molecular, temporal and region-specific requirements of the beta isoform of Calcium/Calmodulin-dependent protein kinase type 2 (CAMK2B) in mouse locomotion. *Sci. Rep.* **6**, 26989; doi: 10.1038/srep26989 (2016).

## Figures and Tables

**Table 1:** Overview of western blot quantification performed on the *Camk2b* mutants in percentage of controls.

	T287A		CAG-CreER							
	Hippocampus		Cortex		Hippocampus		Cerebellum			
Antibody	WT (4)	T287A (4)	Cre− (3)	Cre+(5)	Cre− (3)	Cre+(5)	Cre− (3)	Cre+(5)		
CAMK2B	100 ± 27	84 ± 22	100 ± 16	0 ± 0	100 ± 15	0.2 ± 0.1	100 ± 13	0 ± 0		
CAMK2A	100 ± 24	67 ± 11	100 ± 7	126 ± 25	100 ± 10	89 ± 3	100 ± 13	57 ± 20		
Ph-T287	100 ± 16	0.1 ± 0.1								
Ph-T286	100 ± 11	116 ± 18								

	Δex2									
	Cortex			Hippocampus			Cerebellum			
Antibody	WT (2)	Het (2)	KO (2)	WT (8)	Het (8)	KO (2)	WT (4)	Het (4)	KO (4)	
CAMK2B	100 ± 16	23 ± 8	2 ± 1	100 ± 5	13 ± 2	10 ± 4	100 ± 5	17 ± 5	6 ± 2	
CAMK2A	100 ± 25	109 ± 35	118 ± 45	100 ± 7	96 ± 13	128 ± 34	100 ± 16	64 ± 21	77 ± 28	

	L7-Cre		GABAa6-Cre		RGS9-Cre		EMX-Cre			
	Cerebellum		Cerebellum		Striatum		Cortex		Hippocampus	
Antibody	Cre− (2)	Cre + (2)	Cre− (5)	Cre + (5)	Cre− (4)	Cre + (4)	Cre− (6)	Cre + (6)	Cre− (6)	Cre + (6)
CAMK2B	100 ± 11	19 ± 3	100 ± 17	9 ± 1	100 ± 12	19 ± 4	100 ± 12	2 ± 1	100 ± 8	0 ± 0
CAMK2A	100 ± 15	30 ± 1	100 ± 9	115 ± 7	100 ± 17	104 ± 15	100 ± 23	92 ± 7	100 ± 8	87 ± 6

	L7-Cre			
	Cerebellum			
Antibody	WT (4)	Cre+(2)	WT (4)	Cre− (2)
CAMK2B	100 ± 23	27 ± 5	100 ± 23	139 ± 15
CAMK2A	100 ± 7	18 ± 1	100 ± 7	61 ± 15

WT = wildtype; Het = heterozygous knockout; KO = knockout. Cre– = Cre-negative *Camk2b* mutant; Cre + = Cre-positive *Camk2b* mutant. Number of samples is depicted in brackets.

**Table 2:** Overview of statistical analysis of rotarod performances and learning of *Camk2b* mutants.

	1 Day Paradigm					
	Effect of Genotype		Effect of Time		Interaction	
Mouse Line	F-value	p-value	F-value	p-value	F-value	p-value
L7-Cre	F_(3.45)_ = 0.292	0.8311	F_(3.45)_ = 19.52	**0.0001**	F_(3.45)_ = 1.554	0.1090
GABAa6-Cre	F_(1.14)_ = 1.388	0.2585	F_(1.14)_ = 2.706	**0.0393**	F_(1.14)_ = 1.273	0.2916
RGS9-Cre	F_(1.12)_ = 2.289	0.1562	F_(1.12)_ = 0.362	0.8348	F_(1.12)_ = 0.993	0.4208
EMX-Cre	F_(1.28)_ = 2.235	0.1461	F_(1.28)_ = 14.14	**0.0001**	F_(1.28)_ = 2.724	**0.0330**
	**5 Day Paradigm**					
	**Effect of Genotype**		**Effect of Time**		**Interaction**	
Mouse Line	F-value	p-value	F-value	p-value	F-value	p-value
L7-Cre	F_(3.45)_ = 1.734	0.1723	F_(3.45)_ = 49.49	**0.0001**	F_(3.45)_ = 1.833	**0.0451**
GABAa6-Cre	F_(1.14)_ = 3.697	0.0751	F_(1.14)_ = 7.707	**0.0001**	F_(1.14)_ = 0.878	0.4828
RGS9-Cre	F_(1.12)_ = 1.314	0.2739	F_(1.12)_ = 2.615	**0.0090**	F_(1.12)_ = 1.119	0.3555
EMX-Cre	F_(1.28)_ = 5.824	**0.0224**	F_(1.28)_ = 36.55	**0.0001**	F_(1.28)_ = 2.276	0.0654

A 2-way repeated measures ANOVA was used to test for performance or learning impairments (for details, see material and methods).
